# “Provider discretionary power practices to support implementation of patient-centered HIV care in Lusaka, Zambia”

**DOI:** 10.3389/frhs.2022.918874

**Published:** 2022-09-14

**Authors:** Chanda Mwamba, Njekwa Mukamba, Anjali Sharma, Kasapo Lumbo, Marksman Foloko, Herbert Nyirenda, Sandra Simbeza, Kombatende Sikombe, Charles B. Holmes, Izukanji Sikazwe, Carolyn Bolton Moore, Aaloke Mody, Elvin Geng, Laura K. Beres

**Affiliations:** ^1^Centre for Infectious Disease Research in Zambia, Lusaka, Zambia; ^2^Department of Public Health Environments and Society, London School of Hygiene & Tropical Medicine, London, United Kingdom; ^3^Department of Medicine, Georgetown University Medical Centre, Georgetown University, Washington, DC, United States; ^4^Washington University School of Medicine, St. Louis, MO, United States; ^5^Department of International Health, Johns Hopkins Bloomberg School of Public Health, Baltimore, MD, United States

**Keywords:** discretionary power, patient-centered care, street level bureaucracy, HIV, Zambia

## Abstract

**Introduction:**

Traditional patient-provider relationships privilege the providers, as they possess the formal authority and clinical knowledge applied to address illness, but providers also have discretion over how they exercise their power to influence patients' services, benefits, and sanctions. In this study, we assessed providers' exercise of discretionary power in implementing patient-centered care (PCC) practices in Lusaka, Zambia.

**Methods:**

HIV clinical encounters between patients on antiretroviral therapy (ART) and providers across 24 public health facilities in Lusaka Province were audio recorded and transcribed verbatim. Using qualitative content analysis, we identified practices of discretionary power (DP) employed in the implementation of PCC and instances of withholding DP. A codebook of DP practices was inductively and iteratively developed. We compared outcomes across provider cadres and within sites over time.

**Results:**

We captured 194 patient-provider interactions at 24 study sites involving 11 Medical Officers, 58 Clinical Officers and 10 Nurses between August 2019 to May 2021. Median interaction length was 7.5 min. In a hierarchy where providers dominate patients and interactions are rapid, some providers invited patients to ask questions and responded at length with information that could increase patient understanding and agency. Others used inclusive language, welcomed patients, conducted introductions, and apologized for delayed services, narrowing the hierarchical distance between patient and provider, and facilitating recognition of the patient as a partner in care. Although less common, providers shared their decision-making powers, allowing patients to choose appointment dates and influence regimens. They also facilitated resource access, including access to services and providers outside of scheduled appointment times. Application of DP was not universal and missed opportunities were identified.

**Conclusion:**

Supporting providers to recognize their power and intentionally share it is both inherent to the practice of PCC (e.g., making a patient a partner), and a way to implement improved patient support. More research is needed to understand the application of DP practices in improving the patient-centeredness of care in non-ART settings.

## Introduction

Health providers traditionally hold power over patients within a patient-provider interaction because they are elevated by specialized clinical knowledge, resource access, and health system policies and procedures that confer authority to providers (1–4). Leveraging gaps and inherent flexibility in health service delivery guidance, public service health providers have opportunities to use their discretion in how they exercise their power during patient interactions in service of either facilitating or frustrating patient experiences of healthcare (5–7). Lipsky coined the term ‘street-level bureaucracy' (SLB) to describe this phenomenon (7). Lipsky's theory of SLB (7) postulates that frontline workers function as interpreters of policies and have “the power to exercise a degree of discretion over the services, benefits and sanctions” they provide (8). To fill gaps in training, logistical or supervisory support, health providers rely on their own belief, practice, service delivery, professional, and social networks to operationalize and implement health policies (5–8). This policy interpretation and shared meaning enter routine practice within which health providers moderate services, benefits, and sanctions (7–11). This moderation embedded in informality and resource-constraints empowers providers' role as gatekeepers, who can use their discretion to restrict or enhance access (10), putting patients at a disadvantage; however, this does not always have to be the case as healthcare providers can respond to individual patient and contextual needs through innovations that improve both service delivery processes and outcomes (12, 13). It is important to understand how providers utilize their power within a patient-provider interaction to facilitate patient-centeredness in health service delivery.

Health systems increasingly recognize the importance of patient-centered care (PCC) (14). The World Health Organization recommendation for people-centered HIV care and treatment reiterates the importance of improving patients' experience by respecting patient autonomy and ability to choose the best course of action within their socio-cultural context (15). Conceptualizations of PCC demonstrate that it operates through patient-provider micro-interactions, health system structures (meso-level), and the larger socio-cultural context (macro-levels) (13, 14).

People living with HIV experience the requirements of early HIV diagnosis, linkage to care, and lifetime commitment to ART differently depending on their age, gender, sexual identity, and health status, with each step in the HIV care cascade complicated by their personal, professional, and social circumstances (16–21). Traditionally, HIV care and treatment has been top-down and required patient compliance rather than ownership of life-long antiretroviral therapy (ART) (11, 22). As a result, health providers have used verbal and non-verbal communication to direct, proscribe, control, and persuade patients to adopt what health providers consider acceptable and appropriate behavior for ‘healthy subjects' (10, 11, 22–24). In an exercise of power, health providers perceive patients who miss or delay visits as ‘bad' and subject them to punishing enhanced counseling irrespective of previous ART adherence or the circumstances which delayed them (11, 25–27). In other instances, health providers may change patient care practices due to resource constraints, for example, substituting ART regimens due to low or quickly expiring supplies, which may inadvertently increase the burden of treatment for the patient (28). Additionally, standardized treatment protocols may not account for differential needs of women, adolescents, men, the differently abled, and the aged, putting them under undue stress as they navigate their social dependencies and roles to meet stringent health service requirements and multiple, required appointments at varied locations (16–21). Thus, health providers may exert their discretionary power using their best judgement under the prevailing circumstances, but nonetheless deliver sub-optimal services and leave patients dissatisfied with their HIV care (25). In turn, dissatisfied patients exercise their agency to disengage from HIV care, silently transfer to another clinic, or seek alternate treatments, creating space for opportunistic infections (29, 30).

We created a multi-component intervention to improve patient-centered HIV care in Lusaka, Zambia, which promoted shared decision-making, good communication, and welcoming rather than punishing tones and procedures for those re-engaging in HIV care. While providing no definition during training, we introduced the term ‘discretionary power' simply stating that healthcare workers had the power to use their discretion per patients' specific situation without compromising on policies and guidelines. We provided and solicited examples of discretionary power used to better serve patients from trainees on the 1st day of training. On the 2nd day of training held a week later, we invited them to share experiences with their use of discretionary power after being introduced to the concept. We hypothesized that, when supported, health providers would use discretionary power to increase open, positive interactions and innovative and responsive HIV care (6, 11). Nested within a stepped-wedge, hybrid implementation-effectiveness trial of the PCC intervention, this study utilized qualitative methods to assess the exercise of DP as it relates to the implementation of PCC through examining provider choices (actions and inactions) in the patient-provider clinical interaction. Insights into how providers communicate with patients to understand their socio-cultural context and how they leverage discretionary power to alleviate identified challenges can inform future interventions that improve people-centeredness of care.

## Methods

### Study background

The Center for Infectious Disease Research in Zambia (CIDRZ) implemented the ‘*Person-Centered Public Health for HIV Treatment in Zambia'* (PCPH) study, across 24 Ministry of Health (MoH) facilities in Lusaka Province, Zambia, from August 2019 to November 2021, with the aim of improving health care workers' and patient experiences that would then lead to improved service delivery and clinical outcomes, including retention and viral suppression among patients living with HIV. The PCPH intervention included (1) training health care workers in patient-centered care principles and skills (PCC) including communication practices, (2) on-site mentoring of health care workers on application of PCC, (3) measuring the patient experience and feeding it back to health provider's quarterly through data review meetings, (4) in-kind incentives for improved facility-level performance. The PCPH stepped-wedge trial rolled out the intervention across four, 6-month periods with eight new intervention sites in Period 1, four additional intervention sites in Periods two and three, respectively, and eight additional intervention sites in Period 4.

To understand the implementation of PCC communication within the patient-provider interaction, individual-level consultations between HIV care providers and patients were audio recorded for quantitative analysis using the Roter Interaction Analysis System (RIAS) (31). Nested within this, we transcribed the audio recordings to assess provider use of discretionary power in the implementation of patient-centered HIV care using thematic content analysis (32).

### Study population, recruitment, and sampling

HIV providers, including Medical Officers (MO), Clinical Officers (CO), and Nurses, were purposively sampled to achieve balance across cadres within each of the 24 study facilities. The study sought 5 providers per facility with 1–3 consultations recorded per provider per study period, following them from Period 1 through Period 4. HIV providers were made aware of the study opportunity through an announcement at a staff meeting including study sensitization. Interested providers gave voluntary, written consent to participate after the recruitment meeting. Research staff members then returned on an HIV clinic consultation day to record consults given by participating providers. Patients living with HIV who were present on the day when clinical consultation recordings were planned and seeing a consenting provider were informed about the study in the waiting room in ART clinics prior to seeing providers for their visit. PCPH Qualitative Research Assistants (QRAs) working at the public health facilities identified and recruited ART patients queuing to see any of the recruited HIV providers. Eligible HIV clients were those that were: (a) 18 years and above and (b) spoke one of the study languages: Nyanja, Bemba, Tonga, or English, and (c) voluntarily consented to participate in the study. Consenting patients were enrolled sequentially in the order of their consultation appointment until the sample size for their provider was reached.

To assess use of discretionary power in the implementation of PCC, we sampled intervention facilities during the main intervention phase and over time, including consultations recorded during the first study period in which the facility was an intervention site (Periods 1–3), and all intervention facilities in the final period, Period 4.

Three cadres of providers were included in this study in their role as ART providers: Clinical Officers (COs), Medical Officers (MOs) and Nurses. COs are primary health workers who have completed a 3-year post-secondary general medical education and provide the majority of Zambia's healthcare (33, 34). MOs have completed a minimum of 6 years in medical training, are in short supply (33, 34), and the most senior provider cadre. Some Nurses provide ART consultations after The Zambian Ministry of Health introduced a nurse-centered antiretroviral treatment (ART) prescription initiative to train and support nurses in prescribing ART due to physician shortages (33, 34).

### Study procedures and data collection

Patient-provider interactions were audio-recorded using recorders which were positioned discreetly in the HIV clinical consultation rooms. The recorders were turned on by a QRA at the beginning of a consultation between a consenting provider and consenting patient then the QRA exited the consultation room. After the consenting patient exited the consultation room, the QRA turned off the recorder. For the discretionary power analysis, audio recording were transcribed verbatim and simultaneously translated into English, if applicable.

### Data analysis

We applied qualitative thematic content analysis to identify practices of discretionary power (DP) employed in the implementation of PCC and instances of withholding DP. To develop the set of DP practices for which we coded, two independent analysts (CM, LKB) reviewed literature on DP and discussed the conceptual meaning of both DP and PCC. The analysts then read a 15% sample of transcripts to inductively develop a code book of DP practices, with ongoing dialogue and refinement through using coding memos to guide reflection. The final code book was then applied across all transcripts. Differences in coding were resolved through dialogue. The lead author summarized themes and categories, discussing results with the second analyst and study investigators.

## Results

We enrolled 79 health providers (11 MOs, 58 COs and 10 Nurses) between August 2019 to November 2021 from the 24 intervention facilities. During period 4 data collection, 6 enrolled health providers were not available for the study team to capture their follow-up consultation sessions. Transcripts from 194 consultations were included in our analysis; median recorded consultation time was 7 min 29 s (min: 1:54, interquartile range: 5:38–11:19, max: 23:59). Of the interactions, 23 involved MOs, 142 Cos, and 29 Nurses. We compared the use of discretionary power across provider cadres from the intervention facilities.

Our inductive coding of DP practices facilitating patient-centeredness of care showed three central themes, represented below. Themes and supporting quotes are summarized in Table 1. We contrast the use of DP to improve patient-centeredness under each theme by emphasizing both implemented and missed opportunities for clinicians to use DP to improve patient-centeredness that were evident in the data.

**Table 1:** Themes and quotes.

**Themes**	**Sub-themes**	**Quotes**
Narrowing hierarchical distance	Welcoming patients: not universally done, opens dialogue prior to clinical consultation	CO: How are you? Patient: Am fine and how are you? CO: How is home? Patient: Home is fine CO: It's good that you have decided to come here today Patient: Thank you- [CO]
	Inviting introductions: not universally done, recognizes patient and improves accessibility of provider prior to clinical consultation	Nurse: My name is Mr. XXX, I will be the one attending to you today. I have seen that they have written XXX, is that your name? Patient: Yes. Nurse: I am a nurse so feel free. This is a men's clinic, a clinic that attends to men, so we respect you. [Nurse]
	Offered apology	CO: How are you? Sorry I have delayed you, I was checking on another patient. Patient: It is fine thanks, I understand. [CO]
	Language of team work	CO: Okay we will continue to help each other to better your health. [Nurse]
	Ceding power to the patient	CO: So, you want to stay on the old drugs Patient: Yes, but they are refusing me to stay on the old ones CO: Who is refusing you? Patient: The same people giving us the drugs, I told them I don't want to change but they refused CO: They can't refuse you, you have a right to choose it's your body, it's your health and it's your life Patient: I told them I did not want to, but they have refused CO: No, you won't change [CO]
Active engagement of patients as care partners	Patient invited to ask questions	Nurse: Is there anything else you want to ask? Patient: No, I have understood everything you have shared. I do not think there are any questions. Nurse: It is clear? Patient: Yes. Nurse: We are here for you, even as you come through next week, feel free to ask. [Nurse]
	Information sharing to empower patients' participation in decision making over care	CO: Where you told about your test results? Patient: Yes, they did inform me. That it is high. CO: What did they say is high, educate me so I know? Patient: They said blood is high, not too sure but it is high. CO: I appreciate you informing me on that. It means the HIV virus in your body has multiplied. The whole essence of this medication is to prevent the multiplication of the virus. There are other people who tend to experience a rise in the viral load. And I am not saying you do not take your medication, as you have informed me that you take your medication always. Patient: So, what causes the increase in the virus even when a person takes their medication regularly?
		CO: It could be due to the fact that you get sick every now and then, for most people when they fall ill every now and then, their immune system is weakened. For others it is because at some point they stop taking their medication for some time. When this happens, the medication will not work as it is intended. Yet for others, their bodies just stop responding to a type of medication meaning we have to give them a different type so that the immune system can have a boost. Have you understood now? Patient: Yes. [CO]
	Appointment scheduling	CO: On which day of October would you like to come? Patient: The medication that I am getting today is for how many months? Clinical Officer: Three months. Patient: So, my next appointment is in October? CO: Yes. Patient: I need to choose a date? CO: Yes, a date on which you prefer to come. Patient: Let me just take a look at the calendar. [CO]
Exercising flexible work processes and resource distribution	Facilitating patient referrals	Clinical Officer: Since you are already here pass by MCH (maternal and child health), I will tell the person [counselor] to take you to MCH so she can listen to what they will say. Patient: Okay, thank you. [CO]
	Fee exemption	MO: Sometimes when someone has lost a lot of weight we worry because they may have tuberculosis and we do not want to treatment. So here the doctor had written that you will do an X-ray Patient: Is it free? MO: No, it is k20 (1 USD) Patient: I do not have any money with me. Provider: Okay. You will go for the X-ray today. I will ask them to exempt you so we can make sure that you do not have tuberculosis. If the chest is okay, I will put you on medication for preventing tuberculosis. [MO, 21st September 2021]
	Offered to give patients extra drugs	Nurse: So, what you do is when you do not know the time when you will be coming back, it is better you come through to the facility before leaving (travelling out of town) and let us know so that we can give you extra medication. Patient: Okay. Nurse: Then when you go somewhere far and the medication has finished, carry this same card with you, you can go anywhere and get medication [Nurse]
	Giving out personal contact details	Medical Officer: Apart from getting the medication, we will not ignore that complaint (patient complaint) so I am asking you to go to the lab, after that we will see if there is a problem. Since you want to tell your wife about this issue (HIV status), I will give you my number so that when she returns back home from her mother's house you can ask her to talk to me. I will invite her to come here. I will emphasize that she comes here, she should not be scared. HIV is now under control as long as you follow the instructions and lead a healthy life, you can just see how people are living now. Before, when someone is positive, they used to be defeated but these days medication is available [MO]

### Narrowing hierarchical distance

Traditional power dynamics within the health care system preference the provider. Thus, intentional actions by providers to narrow the hierarchical distance between providers and patients is an act of power-sharing. This power-sharing lays the foundation for a positive patient-provider interaction, which is core to the practice of patient-centered care and taking a biopsychosocial perspective to care (35). This power-sharing was the most frequent DP practice in the data practiced the most by nurses, manifesting in multiple applications of DP, highlighted below.

### Welcoming patients

Welcoming patients meant health providers spent some time chatting with patients before beginning the diagnostic process. Some health providers made kind gestures including inviting patients to take a seat, greeting them, and telling them that they were 'welcome' at the health facilities. The greetings were extended to inquire about their family's well-being, and when patients talked about a difficulty they, a spouse, or another family member was having, the health providers took time to listen and offered supportive words or advice to help them deal with the situation, even if it wasn't related to health.

Clinical Officer: How are you?

Patient: I am fine thank you, how are you?

Clinical Officer: How is home?

Patient: Home is fine.

Clinical Officer: What is bothering you?

Patient: There's nothing really bothering me though I had a slight headache yesterday and I think it was because of some issues I had at home but am feeling better today.

Clinical Officer: Are you okay, are you able to talk about those issues? Is it issues with your children?

Patient: One of my children is a drunkard so he got into a fight with my nephew on new year's day so my son was hurt and that made me spend the whole night with him at the clinic. That disturbed me because the wound he had was deep but otherwise he is fine.

Clinical Officer: Sorry, it's good to know that your son is fine and you are also fine now

Patient: Yes, he is fine now, he is a bit swollen but his fine.

[CO]

The welcoming sessions sometimes ended with the health provider and patient sharing a joke and laughing together, or light banter about a general topic such as the weather.

Occasionally, patients were thanked, congratulated, and commended for visiting the health facilities and providers expressed happiness about their next appointment. Other providers sought patient feedback on the service they had received that day.

### Inviting introductions

To put patients at ease, some health providers introduced themselves at the outset of a consultation by revealing their names and positions in the health facility. Despite receiving the patient's name from the patient file, health providers extended introductions to patients by inviting them to introduce themselves as well. Some providers just confirmed the patient's name, while others inquired as to whether or not the patient knew them or had previously dealt with them.

Nurse: My name is Mr. XXX, I will be the one attending to you today. I have seen that they have written XXX, is that your name?

Patient: Yes.

Nurse: I am a nurse so feel free. This is a men's clinic, a clinic that attends to men so we respect you.

[Nurse]

To get patients comfortable chatting and answering personal questions about their health and behavior, some health providers advised patients to ‘feel free' and shared that they ‘respected' their opinions during introductions.

### Offered apology

Rarely, apologies were used to acknowledge and explain service shortcomings. Health providers expressed regret for service issues that resulted in long wait times and failure to process whole blood count and CD4 count testing for patients

Clinical Officer: How are you? Sorry I have delayed you, I was checking that other patient.

Patient: Am fine thanks and I understand.

[CO]

### Language of teamwork

A few Clinical Officers and Nurses used language that emphasized the idea of an equal care partnership, instead of an expert provider / recipient patient relationship. Providers used phrases such as “will continue to help each other…” and “we can educate one another” to ‘recognize' patients and narrow the hierarchical gap.

Conversely, the analysis of patient-provider interactions revealed that some health providers mostly MOs maintained hierarchical discursive patterns, hurrying through the consult, neglecting to greet, welcome or introduce themselves and presumably sticking to prescribed HIV related inquiries.

In one instance health providers imposed a punishment on a patient for missing a previous appointment. The patient was told that because they were late for their appointment, they would be seen at the end of the queue of patients who were waiting to be seen that day, even though the patient arrived early in the day. When the patient informed their Clinical Officer, he agreed to the punishment.

### Active engagement of patients as care partners

Traditionally, the provider-patient dynamic would be that a provider made decisions and a patient was expected to do what the provider said, without discussion or questions. Changing this behavior includes offering the patient time and space to direct the dialogue within a consultation, equipping the patient to exercise agency over their own care, and ceding power in health system engagement or treatment plans directly to the patient. This demonstrates enhancing core dimensions of PCC including patient involvement in care, recognizing the patient as a unique person, patient empowerment, and patient-clinician communication (5).

### Patient invited to ask questions

One of the most commonly coded sub-themes, Nurses and COs encouraged patient participation in discussions and decisions about their treatment by inviting them to ask questions. They told patients to ‘feel free to ask,' stating ‘we are here for you.' One nurse reminded patients that they had the ‘right' to ask questions of their providers. They recommended individuals not just to rely on information from other sources, but to double-check by speaking with health care providers. Medical doctors only did this on rare occasions.

Nurse: Is there anything else you want to ask?

Patient: No, I have understood everything you have shared. I do not think there are any questions.

Nurse: It is clear?

Patient: Yes.

Nurse: We are here for you, even as you come through next week, feel free to ask.

Patient: Okay.

[Nurse]

As a result of the invitations to ask questions, patients asked about care and treatment themes, for example, ‘what happens when you do not take your medication for like 4 days', and ‘If you are not feeling well-and you have a clinic card, do you come straight here (clinician office)'. Women frequently asked questions concerning reproductive health in the context of HIV as illustrated below:

Patient 1: The only question I would ask is concerning giving birth, we hear stories in our communities that once you have HIV and you try to give birth then children will be dying?

[Patient]

Patient 2: I am positive, can my child be born healthy?

[Patient]

In some cases, particularly among the MOs the conventional dynamic of the provider as the ‘knowledge authority' and the expectation placed on patients to follow the provider's decisions was demonstrated in some circumstances. Some providers asked closed questions throughout the interaction to control the engagement.

### Information sharing to empower patients' participation in decision making over care

Some health providers, most commonly COs, often exercised their discretion over time and information to share knowledge with patients to help them gain a better understanding and ‘empower' them to take charge of their own care. This meant offering a level of detail about their health condition and assessments that went beyond short answers to equip the patient with information as a resource. This demonstrates enhancing core dimensions of PCC including patient involvement in care, patient empowerment, patient information, and patient-clinician communication (13, 35).

To keep patients informed, providers took the time to converse with them, volunteering information about conditions, care decisions, and processes. For example, providers explained in non-clinical terms how ART worked in the body, what a viral load measured, different ways ART could be accessed, when patients should expect to draw and receive lab results, what U=U means, and demonstrative of changes related to the global pandemic, details about the COVID-19 vaccine. This information was offered both based on provider judgement and in response to specific patient inquires. When patients inquired, health workers provided answers tailored to their unique needs and concerns. Some patients expressed an awareness of why certain procedures were crucial after hearing the material.

On the other hand, some health providers withheld information to orient patients and clarify treatment processes. They made referrals without first discussing the patient's condition or concerns or assessing the co-incident symptoms.

### Ceding power to the patient

Although uncommon in DP practices, some health providers attempted to share care and treatment decision-making 'powers' with patients through their discursive approaches. They also listened to what the patients had to say and respected their choices. Patients' concerns were conveyed through information exchange, and health providers educated them of their 'rights' to participate in care decision-making.

Demonstrating power transfer, a health provider educated a patient of their power, saying, ‘you have a right to choose, it's your body, it's your health and it's your life' in a case in which a patient was being ordered to switch from one ARV to another within their first-line regime and they were against it, as illustrated below:

Clinical Officer: Did they change your medication?

Patient: No, but they are saying I will change my medication.

Clinical: They will change it today?

Patient: Yes, but I don't want them to change my medication, they said I have to change and will have to start drinking it in the morning.

Clinical: So, you want to stay on the old drugs.

Patient: Yes, but they are refusing me to stay on the old ones.

Clinical: Who is refusing you?

Patient: The same people giving us the drugs, I told them I don't want to change but they refused.

Clinical: They can't refuse you, you have a right to choose it's your body, it's your health and it's your life.

Patient: I told them I did not want to, but they have refused.

Clinical: No, you won't change.

Patient: I told them I don't want to change they said no everyone has to change their medication

Clinical: No, you will not. Okay you can go to the pharmacy and pick up your drugs and also collect medication for your cough.

[CO]

### Appointment scheduling

The vast majority of ‘next appointment dates' were dictated by the providers. However, some COs and nurses explicitly invited patient engagement. Some practices included a provider suggesting a day and asking if patients were ‘comfortable' with the appointment day, while others asked for a suggested ‘suitable' or ‘preferred' day.

Clinical Officer: On which day of October would you like to come?

Patient: The medication that I am getting today is for how many months?

Clinical Officer: 3 months.

Patient: So, my next appointment is in October?

Clinical Officer: Yes.

Patient: I need to choose a date?

Clinical Officer: Yes, a date on which you prefer to come.

Patient: Let me just take a look at the calendar.

Clinical Officer: Do you still have some tablets at home?

Patient: I have about seven remaining.

Clinical Officer: Your next appointment cannot be on the twenty because by then all the tablets will have finished, so I will set your appointment on the 12th of October on a Monday.

Patient: No, I cannot miss work on a Monday.

Clinical Officer: On which day are you off from work?

Patient: On Saturday. I can come on Saturday.

Clinical Officer: Okay, you will come on the 10th.

Patient: Okay.

[CO]

In addition, to limit the number of clinic visits, health staff coordinated some patients' appointment dates for drug pick-ups and laboratory tests such as viral load testing. Some providers advised patients that if something came up during their appointment or if they fell ill and need medical assistance, they may rearrange their appointment day.

Patient participation was not always sought with authoritative power remaining with the health providers on the other hand. They gave instructions, denying patients chance to participate in treatment decisions. This was especially apparent when it came to scheduling clinic appointments and placing emphasis on treatment adherence.

Clinical Officer: Your next appointment is 30th June 2020

Patient: On which day does the 30th fall?

Clinical Officer: It will be on a Wednesday.

Patient: It becomes difficult for me to come here on working days unless Fridays maybe.

Clinical Officer: So, the thing is you need to come and have your CD4 count checked, and we only do that on Wednesday's so that we get your blood samples and send them for testing, we can't change the appointment date to a date that suits you. Kindly wait outside, you will be called to the pharmacy soon.

[CO].

### Exercising flexible work processes and resource distribution

In a few instances, some health providers went above and beyond their routine duties to help patients in practical ways, such as facilitating patient referrals, offering consultations outside of regular appointments, telephone consultations, and providing more drugs to meet patients' needs.

When making referrals, some providers made personal referrals to other health providers or asked for favors by writing to them notes or following up with them to ensure a smooth process for the patient.

Clinical Officer: Since you are already here pass by MCH (maternal and child health), I will ask the counselor to take you to MCH so she can listen to what they will say.

Patient: Okay, thank you.

[CO]

### Fee exemption

In one instance, an MO, concerned about a patient's weight loss, exempted her from the 20 Kwacha x-ray fee to ensure that results were not delayed. After the participant explained that she did not have any money with her, the Medical Officer said, “You will go for the X-ray today. I will ask them to exempt you so we can make sure that you do not have tuberculosis.” This same provider offered personalized services, engaging with the patient's family member at the patient's request:

### Offered to give patients extra drugs

Offers of extra drugs or alternative arrangements such as enabling drug pick-ups by a patient representative revealed discretionary power practices based on efforts to keep patients' adherent to treatment regardless of livelihood or personal circumstances.

Nurse: So, what you do is when you do not know the time when you will be coming back, it is better you come through to the facility before leaving (traveling out of town) and let us know so that we can give you extra medication.

Patient: Okay.

Nurse: Then when you go somewhere far and the medication has finished, carry this same card with you, you can go anywhere and get medication.

[Nurse]

### Giving out personal contact details

Some MOs advanced the notion of discretionary power by providing personalized service by giving personal phone numbers to patients who needed follow-up telephone consultations or guidance for their family members as shown below.

Medical Officer: Apart from getting the medication, we will not ignore that complaint (patient complaint) so I am asking you to go to the lab, after that we will see if there is a problem. Since you want to tell your wife about this issue (HIV status), I will give you my number so that when she returns back home from her mother's house you can ask her to talk to me. I will invite her to come here. I will emphasize that she comes here, she should not be scared. HIV is now under control as long as you follow the instructions and lead a healthy life, you can just see how people are living now. Before, when someone is positive they used to be defeated but these days medication is available.

[MO]

We found some missed opportunities for health providers to exercise flexibility when patients tried to negotiate for care services and support beyond the routine delivery of care services, but the health providers did not adapt or respond to meet those needs. Some patients requested for different appointment dates, increased drug refill quantities and collection of drugs by another person due to work commitments. However, the health providers declined these requests, reminding patients condescendingly ‘to be grateful for the free medication' as remarked by one Clinical Officer.

Clinical Officer: Even the government is providing this for free, which means you wouldn't have been able to get it if you didn't have money, so you should feel better.

[CO]

When comparing the types of DP practices used by the three health cadres, it was found that nurses consistently sought to reduce the hierarchical distance between them and their patients by welcoming patients and inviting introductions, followed by COs and MOs, who focused on the standard HIV treatment protocol. Both nurses and COs engaged patients as care partners inviting questions and information sharing while MOs facilitated resource distribution and provided personal contact information to support patients outside of usual working hours.

## Discussion

Through our analysis of patient-provider clinical interactions, we found that providers primarily utilized the power at their discretion to implement more patient-centered HIV care by narrowing the hierarchical distance between themselves and patients and engaging patients as partners through inviting patient questions and sharing information. While these are less traditional demonstrations of discretionary power (1, 5, 10) the time, provider manner, and information resources associated with these acts are very much at the discretion of the provider. Consistent with Lipsky's definition of SLB, providers can follow HIV guidelines while maintaining a hierarchy, but the choice to promote equality and patient empowerment has been associated with improved HIV outcomes (5, 7, 14). Attention to these power dynamics in the implementation of PCC is particularly important because, while these were the most frequent, they were not universal, even within these facilities where an intervention to improve patient-provider interactions and PCC was ongoing.

Information sharing and inviting patients to ask questions may play a particularly prominent role in empowering patients to participate in decision making, engage as care partners with health providers and foster agency and self-efficacy over their own HIV care (35–37). Our findings suggest that inviting patients to ask questions is a specific use of the time and avenue to provide information at their discretion to improve an interaction and work toward establishing a relationship with a patient (24, 37). These findings concur with extant literature (1, 23, 38) and a Nurse provider in our sample who directly educated patients on their right to ask questions of providers, that beyond providing training in PCC to providers, mechanisms to teach patients of their rights to participate in the patient-provider relationship may enhance PCC (23, 38). Lipsky recommends supporting front line workers through “ongoing processes of supportive criticism and inquiry to take ownership” (7). In this context, ownership of PCC principals and so exercise their discretionary power in pursuit of PCC goals and to include these power-sharing indicators into performance evaluations as health providers often change their behavior to reflect what is being measured (7).

We also noted interactions that had limited information with no opportunities for asking questions presented to patients (1). Changes in the type of information provided, the amount of information provided, and the language used to give information are all required (39). Patients require information that they can comprehend to exercise critical judgment in decisions about their health care (23, 39). Therefore, providers need to be proactive in facilitating these patient empowerment processes. The first step in doing this is for providers to acknowledge patients as partners, through ceding some power to patients to give them ‘courage' in having a say in their HIV care and developing a positive relationship with patients (1, 23, 40, 41). In a study among Nurses, they argued that they had no time to share information or answer questions (1). However, patients may negotiate their relationship with health providers until a mutually satisfying relationship is reached (23, 39). The Clinical care protocols provide a benchmark for health providers against which to deliver quality care with patients however, implementation in resource limited settings may shape the discretionary application of decisions and actions to manipulate the system to meet the needs of patients (7, 9, 12).

Although operating within policy frameworks and rules, findings highlight that there were few cases when providers exercised discretion to offer flexible services such as agreeing on dates when patients would attend a next appointment or providing patients with contact numbers to foster phycho-social and emotional support for patients and their family. However, their mere existence demonstrates the possibility of applying them to implement PCC and suggests that further work is needed to increase the application of these practices, or to reduce the provider-level discretion involved in their application (9). Usually, policy-practice divergence occurs as a result of the flexibility with which implementers interpret policy and make decisions without their involvement in ‘higher level' policy formulation and its implementation (5, 7, 9). For example, while choosing a differentiated service delivery (DSD) model that improves patient HIV care access likely requires provider time and openness to patient preferences, a policy that providers must ask patients if the reason they missed an appointment was related to an inflexible appointment schedule and, if so, offer DSD models may improve PCC without relying on provider discretion (42). In Zambia, patients recognized that while health providers care for their well-being, ‘they care rudely' by shouting at patients, shutting down questions and conversations, and using their discretionary powers without considering patient needs, for example, exercising inflexible opening, break and closing hours and thereby prolonging wait times in overcrowded non-private conditions (25). In another study in Zambia about patient preferences, patients demonstrated strong preferences for kind vs. rude health providers (43). While there have been calls for health providers to provide ‘patient-centered' care by exercising ‘flexibility' to meet patients' varying needs and circumstances (25), evidence suggests that current HIV care systems and models are not sufficiently client-centered to ensure agility and adaptability to client circumstances (44, 45). In an analysis to re-orient the South African health system toward public health goals and patient centered care highlighted challenges related to dispersed accountability, complex rules and hierarchical procedures (12).

The power differentials between health professionals are determined by the hierarchy of health professionals, which influences the use of discretionary power practices in patient engagements (46–48) and could facilitate the delivery of inflexible care practices. In the hierarchy of the study health providers, MOs with the traditional senior positions have the most power and autonomy followed by the COs, and then nurses who have relatively less power in organized health care (33, 34, 49). These power dynamics may affect the strategic choices made by each health professional about whether to narrow the hierarchical distance with patients and engage patients as care partners, and these decisions directly influence patient experiences (46–48). For example, we found that power-sharing procedures were largely practiced by nurses in this study, but MOs rarely attempted to share decision-making power with patients or facilitate direct patient participation in care decisions. This suggests that work culture change based on front-line provider choices should be nurtured through training, sensitization, and support for providers in adopting patterns of practice, routines, and policy interpretations to deal with service dynamics (50–53). These changes could, for example be facilitated by new forms of leadership that enable sensemaking in support of building patient-provider relationships and inter-professional collaboration across organizational boundaries (12, 50–53). Leadership teams have a vital role to play in aligning values, fostering employee relationships, and supporting shared understanding of work culture transformation and mutual accountability (50–53). The impact that such effects can have on the patient care experience are well-known (54, 55) including improved patient education, treatment adherence, and self-care on the part of patients, all of which lead to improved health outcomes (50–55).

### Limitations

The estimated patient-provider interaction time is based on the length of the interaction recording. This may over-estimate the actual interaction time, as it may include interruptions, such as a provider being called out of the consultation room, that were not accounted for in the record of the interaction length. With regards to the providers, not all of them included were trained in PCC during the study implementation as the training took a facility approach involving different teams to improving PCC. Another limitation is that the analysis did not compare DP practice in the control facilities and focused on provider behavior in intervention facilities. Further, patient experiences were not explored to understand whether they were satisfied with the provider's discretionary use of power. However, the findings are useful in that they can be used to inform about the use of discretionary power among providers in health settings and how this affects delivery of PCC.

### Future research

To better understand the dynamics of DP practice, more research into the relationship of providers to the practice of different DP techniques would be beneficial. For example, why were nurses able to be more welcoming than MOs and COs. This information could help to unravel health care system complexities that could influence provider policy implementation, as well as serve as a foundation for improving cadre in needed areas, changing the structure or organization of work to improve information sharing and discretionary use of time, and taking into account power dynamics, all with the goal of improving patient care.

## Conclusion

To promote the implementation of patient-centered care, it is critical to understand how health providers exercise DP to support or impede a patient-centered care experience. Health systems can support patient-centered care within patient-provider interactions by training and mentoring providers to share power with patients by narrowing the hierarchical distance between them and patients, sharing information and engaging patients as partners, and offering flexible services, all of which are inherent in the practice of patient-centered care.

## Data availability statement

The datasets presented in this article are not readily available because the Government of Zambia allows data sharing when applicable local conditions are satisfied. To request data access, contact the Secretary to the CIDRZ Ethics and Compliance Committee/Head of Research Operations, Hope Mwanyungwi, mentioning the intended use for the data. Requests to access the datasets should be directed to Hope.Mwanyungwi@cidrz.org.

## Ethics statement

The studies involving human participants were reviewed and approved by the University of Zambia Biomedical Research Ethics Committee (UNZABREC), the University of Alabama at Birmingham (UAB), and the National Health Research Authority and the Zambian Ministry of Health. The patients/participants provided their written informed consent to participate in this study.

## Author contributions

CBM, LB, EG, AS, and AM supported conceptualization. CM, NM, KL, MF, HN, SS, and KS supported data collection. CM and LB conducted formal analysis. CM, LB, AS, NM, EG, AM, IS, and CBM contributed to data interpretation. IS, CBM, CH, and EG acquired study funding. AS, KS, CH, IS, CBM, AM, EG, and LB were study investigators. CM, LB, AS, NM, AM, and EG designed the methodology. SS, KS, NM, and CM conducted project administration. CM, NM, AS, and LB wrote the original manuscript draft. All co-authors reviewed and edited the final draft. All authors contributed to the article and approved the submitted version.

## Funding

This research was supported by the Bill and Melinda Gates Foundation grant number INV-010563.

## Conflict of interest

The authors declare that the research was conducted in the absence of any commercial or financial relationships that could be construed as a potential conflict of interest.

## Publisher's note

All claims expressed in this article are solely those of the authors and do not necessarily represent those of their affiliated organizations, or those of the publisher, the editors and the reviewers. Any product that may be evaluated in this article, or claim that may be made by its manufacturer, is not guaranteed or endorsed by the publisher.
